# Mechanoporation enables rapid and efficient radiolabeling of stem cells for PET imaging

**DOI:** 10.1038/s41598-022-06938-6

**Published:** 2022-02-22

**Authors:** Kyung Oh Jung, Ashok Joseph Theruvath, Hossein Nejadnik, Anna Liu, Lei Xing, Todd Sulchek, Heike E. Daldrup-Link, Guillem Pratx

**Affiliations:** 1 Division of Medical Physics, Department of Radiation Oncology, School of Medicine, Stanford University, Stanford, CA 94305 USA; 2 Molecular Imaging Program at Stanford (MIPS), Stanford University, Stanford, CA 94305 USA; 3 Department of Anatomy, College of Medicine, Chung-Ang University, Seoul, Korea; 4 Molecular Imaging Program at Stanford, Department of Radiology, Stanford University, Stanford, CA 94305 USA; 5 Department of Biomedical Engineering, Georgia Institute of Technology, Atlanta, GA 30332 USA; 6 Department of Pediatrics, Stanford University, Stanford, CA 94305 USA; 7 Department of Radiology, University of Pennsylvania, Philadelphia, PA 19104 USA

**Keywords:** Biochemistry, Biotechnology, Cell biology, Chemical biology, Molecular biology, Stem cells

## Abstract

Regenerative medicine uses the patient own stem cells to regenerate damaged tissues. Molecular imaging techniques are commonly used to image the transplanted cells, either right after surgery or at a later time. However, few techniques are fast or straightforward enough to label cells intraoperatively. Adipose tissue-derived stem cells (ADSCs) were harvested from knee joints of minipigs. The cells were labeled with PET contrast agent by flowing mechanoporation using a microfluidic device. While flowing through a series of microchannels, cells are compressed repeatedly by micro-ridges, which open transient pores in their membranes and induce convective transport, intended to facilitate the transport of ^68^Ga-labeled and lipid-coated mesoporous nanoparticles (MSNs) into the cells. This process enables cells to be labeled in a matter of seconds. Cells labeled with this approach were then implanted into cartilage defects, and the implant was imaged using positron emission tomography (PET) post-surgery. The microfluidic device can efficiently label millions of cells with ^68^Ga-labeled MSNs in as little as 15 min. The method achieved labeling efficiency greater than 5 Bq/cell on average, comparable to 30 min-long passive co-incubation with ^68^Ga-MSNs, but with improved biocompatibility due to the reduced exposure to ionizing radiation. Labeling time could also be accelerated by increasing throughput through more parallel channels. Finally, as a proof of concept, ADSCs were labeled with ^68^Ga-MSNs and quantitatively assessed using clinical PET/MR in a mock transplant operation in pig knee joints. MSN-assisted mechanoporation is a rapid, effective and straightforward approach to label cells with ^68^Ga. Given its high efficiency, this labeling method can be used to track small cells populations without significant effects on viability. The system is applicable to a variety of cell tracking studies for cancer therapy, regenerative therapy, and immunotherapy.

## Introduction

Stem and progenitor cells have demonstrated significant promise for clinical use in regenerative medicine^1^. These therapies can reduce the pain from cartilage deterioration in patients suffereing from joint disease by replacing and repairing cartilage and stimulating bone formation^2^. However, it is necessary to distinguish the transplanted cells from host cells in living subjects and monitor their implantation, survival, migration, and differentiation to predict the therapeutic efficacy^3,4^.

In vivo molecular imaging tools can be used to study cell trafficking in physiological and pathological processes^5^. Many methods are available to label cells ex vivo and image their distribution in vivo^6^. Indirect labeling approaches use reporter genes to transfect cells and allow their visualization in vivo. Direct cell labeling approaches tag cells with contrast agents and nanoparticles using a variety of approaches such as passive incubation with liposome-based transfection agents and electroporation. Regardless of the labeling method, labeled cells can be transplanted and imaged non-invasively in living subjects using fluorescence, bioluminescence, single-photon emission computed tomography (SPECT), positron emission tomography (PET), or magnetic resonance imaging (MRI)^7^. Among all these modalities, PET is unique due to its exceptional sensitivity, sufficient to detect picomolar level of probe in humans. For this reason, it has been employed in various cell tracking applications in vivo^8,9^. In fact, we found that PET was even sensitive enough to image and track single cells in mice^10^. In vivo molecular imaging is therefore essential to track transplanted therapeutic cells and improve our understanding of tissue regeneration^11^.

Ideally, the harvest of therapeutic stem cells and their transplantation for regenerative therapy is conducted within a single operation^1,2^. Therefore, any procedure performed on the transplanted cells, including cell labeling, must be performed within a short time while the patient is being operated^12,13^. In a previous study, we demonstrated that harvested ADSCs could be co-labeled with iron oxide nanoparticles and ^18^F-FDG through a novel microfluidic device, allowing for nearly instantaneous cell labeling and tracking using PET/MRI^14^. The microfluidics device is designed to apply gentle cell compression (also known as mechanoporation) that opens transient pores in the cell membrane and enables convective transport of contrast agents into the cytosol^15,16^. Recently, the technology has been demonstrated for transfection of mRNA into human primary cells^17^. As further application of this microfluidics technology, we here demonstrate a variation of this technique for stem cell imaging using mesoporous silica nanoparticles (MSNs). MSNs have received considerable attention in the field of nanomedicine due to their many attractive features such as high surface area, large pore volume, tunable pore diameter, and narrow pore size distribution^18,19^. In addition, MSNs can efficiently ferry large amounts of molecular cargo, including drug and small biogenic molecules, into cells^20,21^. Using this property, we previously used MSNs to efficiently label cells with radiometals such as ^68^Ga and ^89^Zr through passive incubation and thereby achieve single-cell sensitivity in vivo^10^. However, this approach was relatively slow and could result in detectable toxicity due to the long incubation with radioactive compounds during the incubation phase.

**Figure 1:**
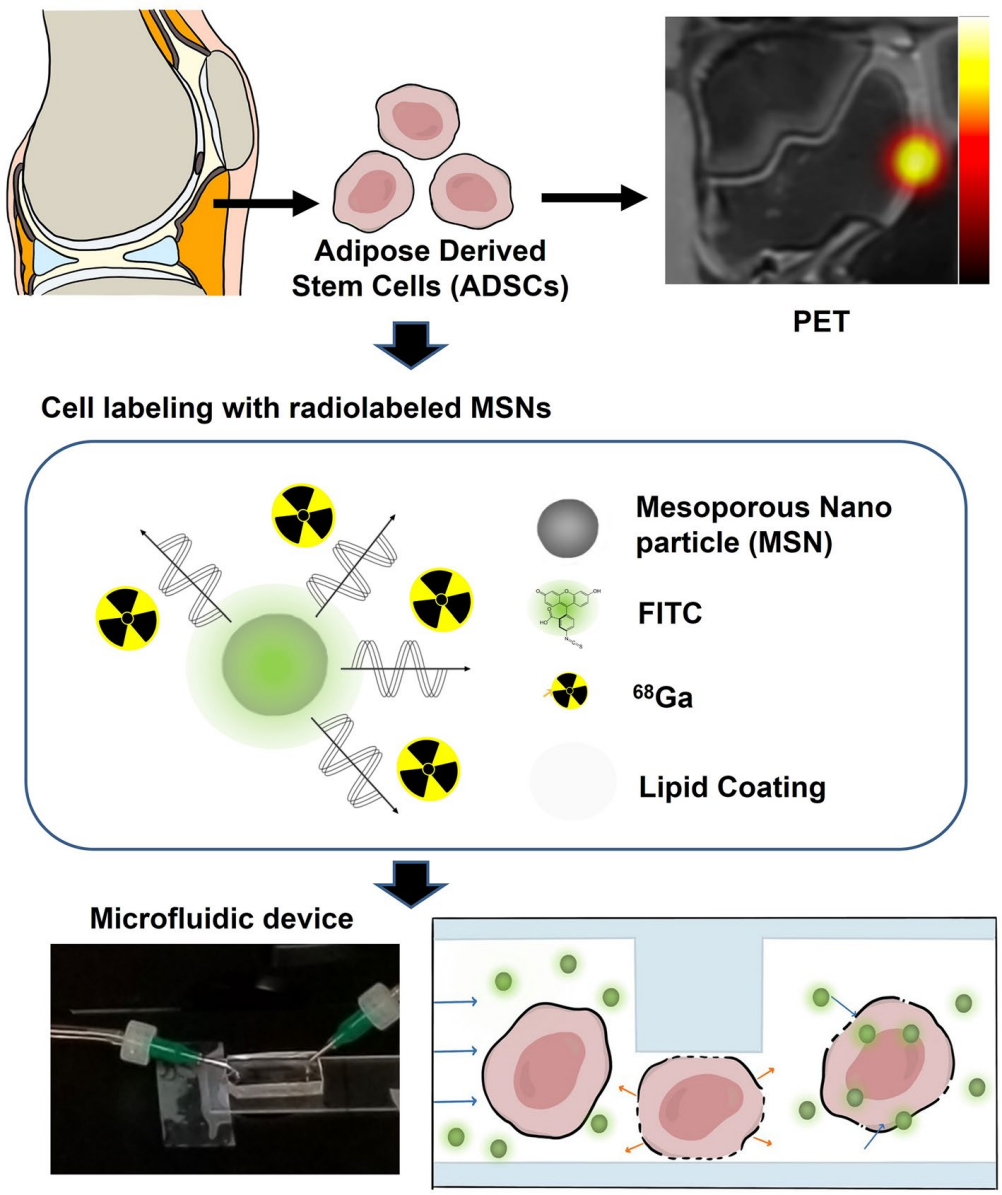
Experimental scheme. Adipose-derived stem cells (ADSCs) were harvested from knee joints of Goettingen minipigs. For PET imaging, ADSCs were labeled by radiolabeled MSNs with ^68^Ga. Through a microfluidic device, approximately 40% compression of ADSCs was achieved to promote transport of radiolabeled MSNs into cells. Finally, the engraftment of labeled cell in knee joints was imaged using PET.

The purpose of this study was to investigate mechanoporation to nearly instantly label therapeutic cells with ^68^Ga-labeled MSNs (Fig. 1) and measure its labeling efficiency compared to conventional co-incubation. The radiolabeling procedure is rapid and straightforward to implement, therefore ideal for regenerative medicine applications that require intraoperative cell labeling. With its short half-life and increasing clinical availability, ^68^Ga permits sensitive and quantitative verification of cell implantation shortly after surgery, while having negligible biological effect on the implanted cells and the patient.

## Materials and methods

### Microfluidic device

A customized microfluidic device was designed to label cells with high throughput using mechanoporation. The device comprises one inlet, 3 or 5 mechanoporation channels in which chevron ridges (9.6 µm gap size) were embedded, and one outlet^14^. The devices were fabricated using standard polydimethylsiloxane (PDMS) molding procedures. Then a plasma bonder (PDC-32G Harrick) was used to bond to microscope glass slides with PDMS.

### Cell culture

All experimental procedures involving animals comply with the ARRIVE guidelines. In addition, our study protocol was approved by the Administrative Panel on Laboratory Animal Care of Stanford University. All experiments involving animals were performed according to the approved protocol. Adipose tissue-derived stem cells (ADSCs) were isolated and harvested from the knee joints of Goettingen minipigs (Marshall Farms, North Rose, NY). According to our previously established techniques^14^, tissue samples were collected from the infrapatellar fat pad, dissociated with type I collagenase, and the isolated cells were characterized based on specific stem cell markers. The cells were cultured in Dulbecco's Modified Eagle Medium (DMEM) medium supplemented with 1% antibiotic–antimycotic mix and 10% fetal bovine serum (FBS). For experiments, ADSCs were expanded up to passage 6.

### Characterization of MSNs by TEM

Propylamine-functionalized mesoporous silica nanoparticles (200 nm particle diameter and 4 nm pore size, Sigma-Aldrich, St Louis, MO, USA) were deposited on carbon/formvar coated copper grids and the size of particles were measured by TEM (Transmission Electron Microscope, JEM-1400 series 120 kV, JEOL USA Inc., Pleasanton, CA, USA). Cells labeled thorugh mechanoporation were fixed with 2% glutaraldehyde and imaged using the same procedure.

### Radiolabeling of MSNs with ^68^Ga

To label MSNs with ^68^Ga, a chelator-free reaction method was used^10,22^. This approach results in the formation of a stable coordination complex between the radiometals and deprotonated Si–O–groups on the MSN surface First, 4.5 µg of MSNs, activated in ethanol overnight, were mixed with 1.5 ml of ^68^GaCl_3_ solution (~ 600 MBq) eluted with HCl (0.1 N), 30 µl of amonium hydroxide solution (Sigma-Aldrich, St Louis, MO, USA), and 100 µl of MES buffer (0.1 M, pH 7.3) to maintain a reaction pH of 7.3. The mixture was incubated at 75 °C for 15 min, and then centrifuged at 14,000 rpm to remove residual ^68^Ga. Radiolabeling purity was analyzed using an AR-2000 radio-TLC plate reader (BioScan Inc., Washington, DC, USA). Before cell treatment, the MSNs were coated with a lipid bilayer using cationic liposome transfection agent (Lipofectamine 2000, Invitrogen, California, USA), as previsouly described^10^.

### Fluorescence imaging and flow cytometry

For in vitro validation, MSNs were labeled with FITC (fluorescein isothiocyanate isomer I; Sigma-Aldrich, St Louis, MO, USA) by mixing 1 mg of MSNs with 0.1 mg of FITC in 1 ml volume. After incubation at room temperature for 1 h, the mixture was washed with PBS to remove excess FITC. For cell treatment, a cationic liposome transfection agent (Lipofectamine 2000, Invitrogen, California, USA) was used to coat MSNs with a lipid layer. For mechanoporation cell labeling, ADSCs (2 × 10^5^ cells) were mixed with 1 ml lipid-coated FITC-MSNs (4.5 µg/ml) in 2 ml FACS buffer [3x] and 3 ml PBS and passed through the microfluidic device (flow rate 0.5 ml/min). For microscopic visualization, cells were labeled with a fluorescent membrane tracer, DiI (Invitrogen, Carlsbad, CA, USA; red fluorescence; Ex: 565 nm, Em: 594 nm; incubation at 37 °C for 15 min), and a nucleus stain, Hoechst 33,342 (NucBlue Live, ReadyProbes; Thermo Fisher Scientific, Waltham, MA, USA; incubation at 37 °C for 5 min). To confirm the uptake of FITC-MSNs, cells were visualized by fluorescence microscopy (EVOS FL, ThermoFisher Scientific, Santa Clara, CA, USA) and quantified by flow cytometry (BD FACSAria Fusion sorter, BD Bioscience, San Jose, CA, USA).

### In vitro PET imaging and gamma counting

ADSCs (2 × 10^5^ cells) were radiolaeled by mixing them with 1 ml ^68^Ga-MSNs (~ 25 MBq/ml) in 2 ml FACS buffer [3x] and 3 ml PBS and passing them through the mechanoporation device (5 channels, flow rate 0.5 ml/min). After mechanoporation, the cells washed three times with PBS to remove residual ^68^Ga-MSNs, then the labeled cells (3–48 × 10^3^ cells) were seeded in 24 well plate for PET imaging. For comparison, ADSCs were also passively incubated with ^68^Ga-MSNs (~ 25 MBq/ml for 10, 30, and 60 min) and seeded in the same plate. The well plate was imaged using PET (10 min scan; Inveon D-PET, Siemens Preclinical Solutions, Knoxville, TN). For quantitation, region of interest (ROI) analysis was performed on PET images using the Inveon Research Workplace (IRW) software. After PET imaging, the absolute radioactivity of cells was confirmed by gamma counting (AMG, Hidex, Turku, Finland). We also assessed radiolabel efflux by labeling ADSCs (1 × 10^4^ cells/well) with ^68^Ga-MSNs using the microfluidics device, then incubating the cells for 30, 60, 90, or 120 min in 24 well plate. After centrifugation and washing in PBS, the remaining radioactivity was measured by gamma counting.

### Cell viability assays

To assess the potential toxicity of the labeling procedure, labeled and unlabeled ADSCs (2.5 × 10^3^ cells/well) were seeded in 96-well plates and incubated for 48 h. The cells were then incubated with CCK-8 solution (Sigma-Aldrich, St Louis, MO, USA) for 1 h. The mean optical density (OD) of the samples was measured at 450 nm using a GloMax multi-detection system (Promega, Madison, WI, USA). In addition, DNA damage was assessed using the γH2AX assay. ADSCs (1 × 10^4^ cells/well; either unlabeled, or labeled using mechanoporation or 60 min passive incubation) were seeded and cultured for 1 h in Lab-Tek II chamber slides (ThermoFisher Scientific, Santa Clara, CA, USA). After one hour, these cells were fixed in 4% formaldehyde for 10 min and stained using primary anti-phospho-histone H2A.X (Ser139) antibody (1:100; cat# 05-636, Sigma-Aldrich, St Louis, MO, US). After overnight staining at 4 °C, secondary staining was conducted using anti-mouse Alexa Fluor 488 antibody (1:100; ThermoFisher Scientific, Santa Clara, CA, USA). Then, the stained cells were visualized by fluorescence microscopy (EVOS FL, ThermoFisher Scientific, Santa Clara, CA, USA) and the images were quantified using Image J software. Additionally, apoptosis was detected using an Annexin-V staining kit (Abcam, Cambridge, England). ADSCs (1 × 10^5^ cells/well) were seeded and cultured for 48 h in 24-well plates post labeling. Then, the stained cells were visualized by fluorescence microscopy and the images were quantified using Image J software. Finally, to assess proliferation, ADSCs (1 × 10^4^ cells/well) were seeded and cultured for 48 h post-labeling in Lab-Tek II Chamber SlideTM (1:100; ThermoFisher Scientific, Santa Clara, CA, USA). After 48 h, these cells were fixed in 4% formaldehyde for 10 min, and stained using primary anti-Ki67 antibody (1:100; cat# ab15580, Abcam, Cambridge, UK). After overnight staining at 4 °C, secondary staining was conducted using using anti-rabbit Alexa Fluor 594 antibody (1:100; ThermoFisher Scientific, Santa Clara, CA, USA). The cells were then visualized by fluorescence microscopy and the images were quantified using Image J software.

### PET/MR imaging of dual-labeled stem cells in pig knee joints

To evaluate the approach in a clinical environment, we used a previously established model of cell transplantation based on artificially created cartilage defects in pig knee joints^22^. ADSCs were dual-labeled with ^68^Ga-MSNs and Ferumoxytol (AMAG Pharmaceuticals Inc., Cambridge, MA, USA), used here as an MRI contrast. For Ferumoxytol, a FDA-approved iron supplement, we followed the labeling protocol from our previous study^14^. First, ADSCs (1 × 10^7^ cells) were mixed with Ferumoxytol (10 mg/ml) and ^68^Ga-MSNs (~ 100 MBq/ml) in FACS buffer and PBS, then passed through the microfluidic device (5 channels, flow rate 0.5 ml/min). After mechanoporation, the cells were washed 3 times with PBS to remove residual Ferumoxytol and ^68^Ga-MSN. Dual-labeled (*n* = 4) and unlabeled (*n* = 4) cells were implanted into 8 cartilage defects in the femoral end of pig knee specimens. The cells were implanted using fibrin glue (Ethicon, Somerville, NJ) and then the joint capsule, muscles and skin were sutured. PET/MRI images were obtained using a clinical 3 T Signa PET/MR scanner (GE Healthcare, Milwaukee, Wisconsin). PET images were acquired using a 30 min acquisition time, simultaneously with the MRI acquisition. MRI included proton density (PD) weighted fast spin echo (FSE) with fat saturation [acquisition time (TA) = 16 min, field of view (FOV) = 14 cm, repetition time (TR) = 2700 ms/echo time (TE) = 32 ms, flip angle (FA) = 110°, matrix size 192 × 192, slice thickness (SL) = 1 mm] and multi-echo spin echo sequences (TR = 1200, TE = 10,20,30,40,50,60,70,80, FA = 90, matrix size 192 × 192, slice thickness 1.1, FOV = 14, TA = 13 min). To quantify the PET images, the scanner specific software (Image QC, GE Healthcare, Milwaukee, Wisconsin) was used. To quantify the MRI images, T2-relaxation time maps were generated and T2-relaxation times of each implant were measured.

### Statistical analysis

All data are presented as mean ± standard deviation. Statistical significance was considered attained for *P* values < 0.05 based on Student’s *t*-test (two-tailed, unpaired samples).

## Results

### MSN characterization and cell uptake

**Figure 2:**
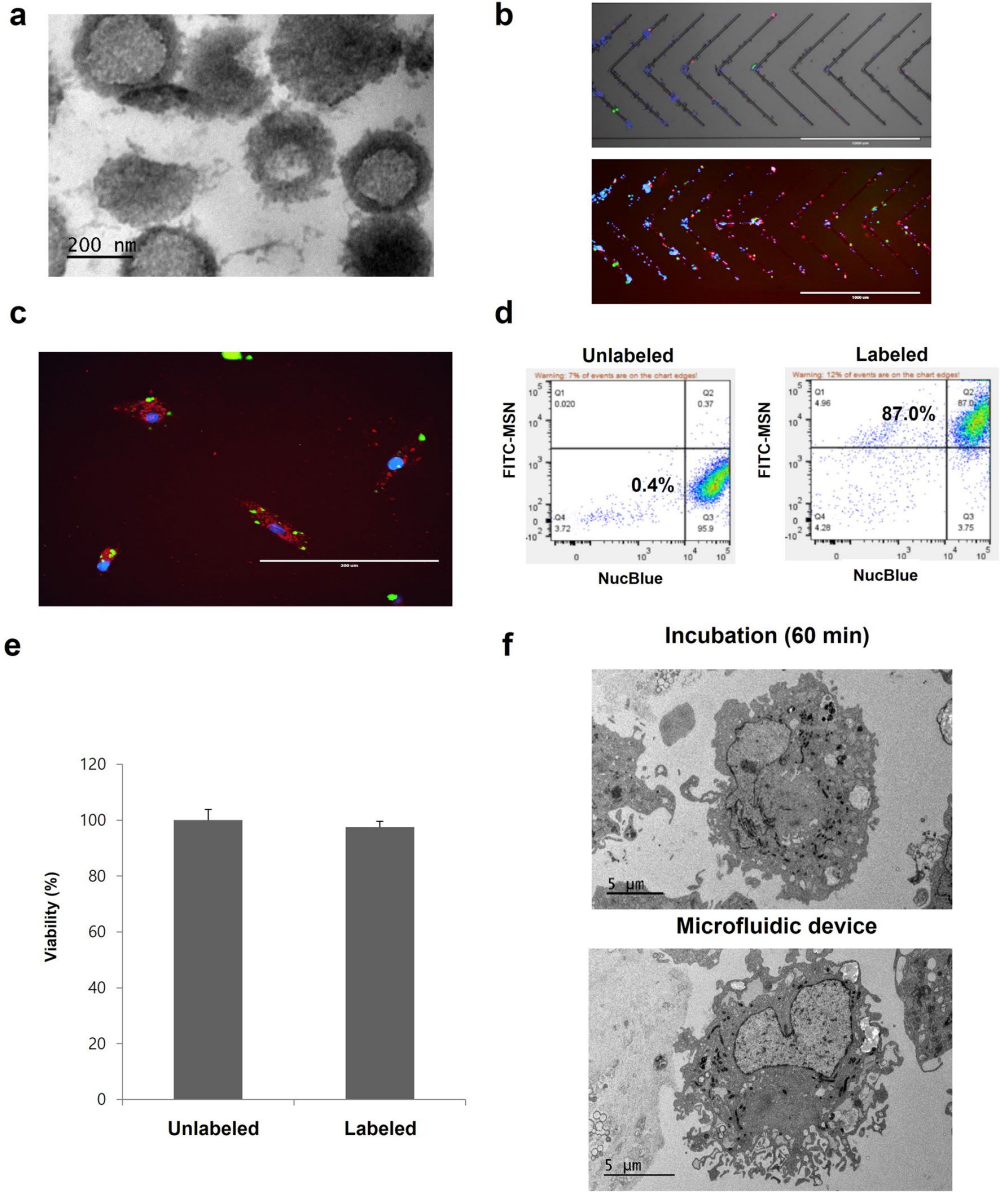
Demonstration of cell labeling using mechanoporation. (**a**) TEM showing spherical morphology of MSNs. (**b**) Fluorescence microscopy showing labeling of ADSCs by FITC-MSNs inside mechanoporation device (green:FITC; blue:nucleus; red: cell membrane). (**c**) FITC-MSNs were efficiently transported into ADSCs. (**d**) FACS analysis showing that labeled cells have higher green fluorescence than unlabeled cells. (**e**) CCK viability found no significant difference between unlabeled and labeled cells. (**f**) TEM images showing cellular uptake of MSNs in ADSCs after passive incubation (60 min) and microfluidic mechanoporation (0.5 ml/min).

The structure of the MSNs was characterized using TEM to reveal spherical nanoparticle morphology (50–500 nm diameter) with obvious pore structure (Fig. 2a). Nanoparticle Tracking Analysis (NanoSight Ltd) confirmed a mean particle size of 178 nm with a standard deviation of 83 nm (Supplementary Fig. 1). In addition, according to our zeta-potential measurements, propylamine-functionalized MSNs dispersed in distilled water have on average a slight positive charge. The zeta-potential is 9.75 mV with 11.6 mV standard deviation (Supplementary Fig. 2). After lipid coating with lipofectamine, MSNs displayed increased positive charge, as expected. The zeta-potential is 34.4 mV with 10.1 mV standard deviation. The nanoparticles were then labeled with fluorescent FITC to visualize their internalization by ADSCs during mechanoporation (Fig. 2b). Afterwards, ADSCs demonstrated green fluorescence characteristic of MSN uptake, as well as blue nuclear staining and red cystosol fluorescence from DiI staining (Supplementary Fig. 3). The labeled cells were then seeded in 24 well plates and imaged by fluorescence microscopy to confirm uptake of FITC-MSNs in the cytosol (Fig. 2c). Using flow cytometry, we quantified the uptake of MSNs by ADSCs and found that labeled cells had 87% higher green fluorescence compared to unlabeled cells (Fig. 2d), indicating that use of the microfluidics procedure enables efficient transport of MSNs into cells. There was no significant decrease in cell viability after processing by mechanoporation (CCK-8 assay, Fig. 2e). Finally, using TEM imaging, we confirmed cellular uptake of MSNs into the cytosol following mechanoporation (Fig. 2f).

### Instant stem cells radiolabeling by mechanoporation

**Figure 3:**
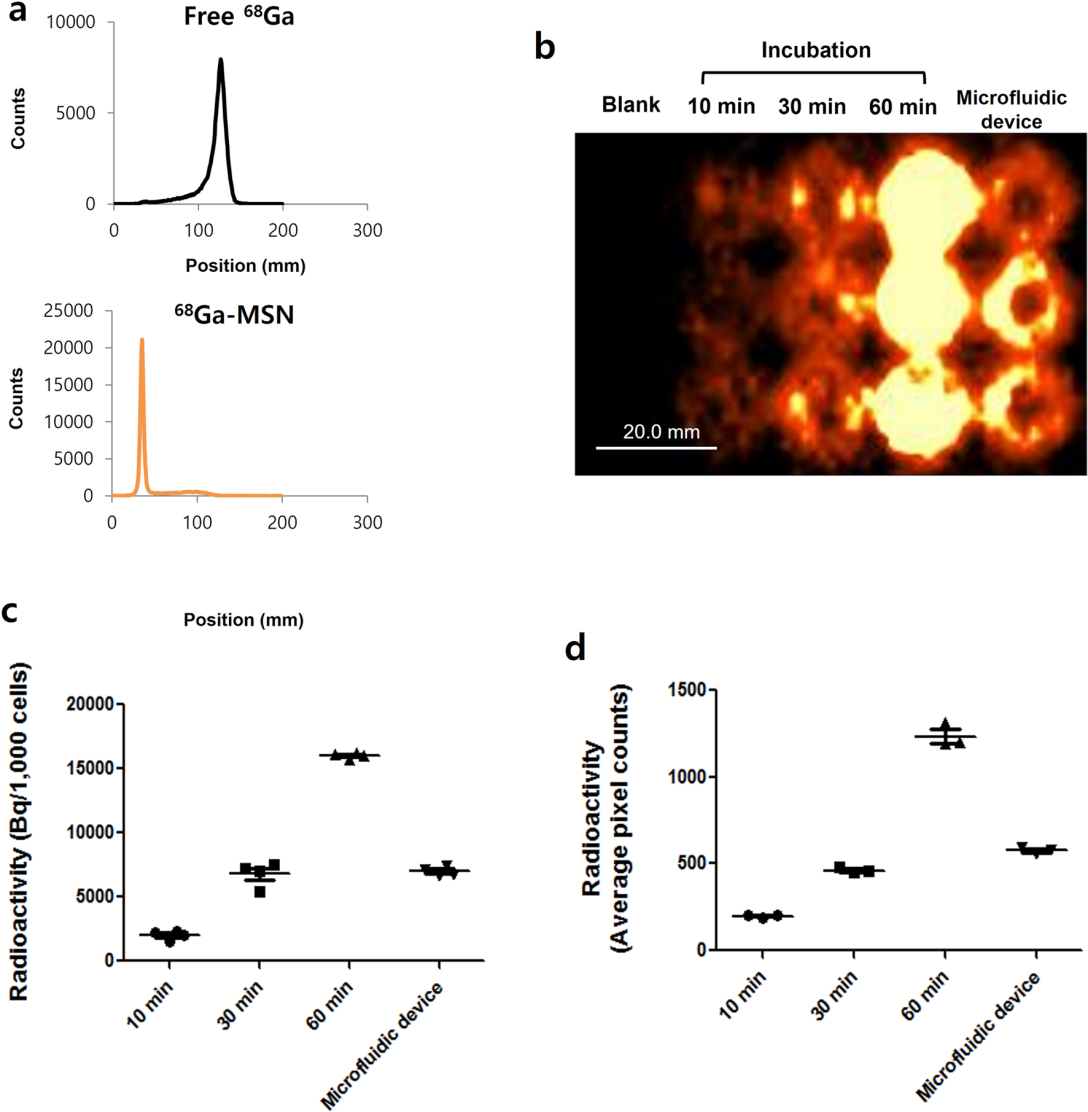
Cell radiolabeling using mechanoporation device. (**a**) Thin-layer chromatography showing efficient ^68^Ga-labeling of MSNs. (**b**) PET images showing comparing cell labeling efficiency for passive transport (10 min, 30 min, and 60 min incubation) and microfluidic mechanoporation and (**c**) region-of-interest quantification of the images. (**d**) Gamma counting of the cell samples.

Cells labeling experiments were repeated using MSNs radiolabeled with with clinical-grade ^68^Ga. Using thin-layer chromatography, we confirmed that the MSNs were successfully labeled with ^68^Ga (Fig. 3a). The microfluidics device was again used to label cells, and took less than 15 min to process 2 × 10^5^ cells at a flow rate of 0.5 ml/min. The radiolabeled cells were then seeded at increasing concentrations in 24 well plates and imaged using PET (Supplementary Fig. 4a). To quantify the uptake in the different wells, we performed region-of-interest (ROI) analysis of the PET images and gamma counter measurements and found a linear increase in sample radioactivity with increasing cell number (Supplementary Fig. 4b, c). Next, we compared labeling efficiency between passive incubation (10, 30, and 60 min) and instant microfluidic-based mechanoporation. Cell uptake was detectable in the PET images for all the different labeling schemes (Fig. 3b). For passive incubation, the intensity of the signal increased with longer incubations. The cells labeled by instant mechanoporation showed intensity similar to cells labeled by passive incubation for 30 min (Fig. 3c, d). Finally, using gamma counting, we measured radiotracer efflux in the labeled cells and found it to reach about 50% after two hours (Supplementary Fig. 5).

### Biocompatibility of cell labeling procedure

**Figure 4:**
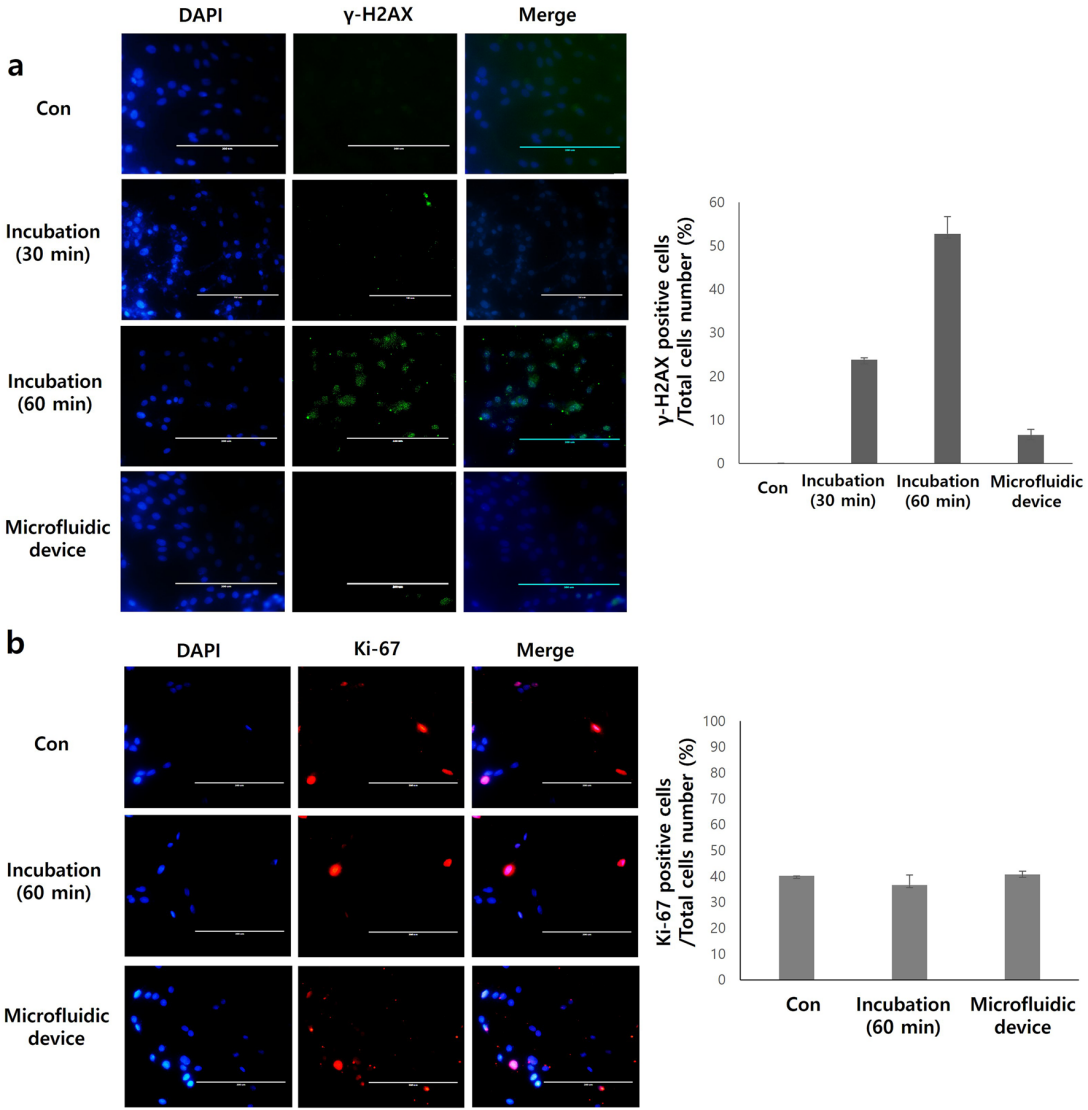
Cell biocompatibility assays. (**a**) γH2AX staining showing DNA damage (double strand breaks) in labeled ADSCs after 30 min, 60 min passive incubation or microfluidic device. (**b**) Ki-67 staining showed no significant difference in proliferation between control, unlabeled and labeled cells.

To confirm the biocompatibility of the labeling procedure, we characterized viability, DNA damage, proliferation, and apoptosis in mechanoporation-labeled cells. First, to assess the acute toxicity of mechanoporation, cells were flowed through the device together with ^68^Ga-labeled MSNs, then trypan blue staining was performed 2 h after the procedure. Based on microscopy visualization, viability is estimated as 100% in unlabeled cells and 94.0% in MSN-treated and mechanoporated cells, suggesting that mechanoporation in itself does not induce acute toxicity (Supplementary Fig. 6). We then assessed long-term toxicity in cells incubated or mechanoporated with ^68^Ga-labeled MSNs. Cells passively incubated for 60 min presented γH2AX foci characteristic of radiation-induced double-strand breaks (Fig. 4a). These foci were not detectable in cells labeled by mechanoporation, suggesting that the speed of the procedure reduced exposure to ionizing radiation and ensuing radiotoxicity. Quantification of γH2AX images found fivefold higher proportion of cells showing DNA damage after 60 min incubation with ^68^Ga-MSN compared to mechanoporation labeling. Next, Ki-67 staining found no significant difference between labeled and unlabeled cells in terms of proliferation (Fig. 4b). The same trend was observed for apoptosis, as no significant difference between the different groups was observed (Supplementary Fig. 7). Overall, these results show that mechanoporation can label cells without detectable increase in long-term toxicity. In fact, compared to passive incubation, mechanoporation resulted in lower DNA damage thanks to the shorter exposure to radioactive MSNs. In addition, since different media were used for passive incubation and mechanoporation, we also confirmed that the use of FACS buffer (diluted with PBS) for mechanoporation did not enhance the uptake of MSNs by the cells. Cells passively incubated in either culture medium or FACS buffer took up similar amounts of MSNs (Supplementary Fig. 8), indicating that the increased uptake seen in the mechanoporated cells was due to the action of the device and not the use of a different buffer.

### PET/MRI imaging of dual-labeled stem cells in pig knees

Finally, we performed a mock cell transplant operation under PET/MRI image guidance. The stem cells were dual-labeled with a 50:50 mixture of radiolabeled MSNs and iron oxide nanoparticles. After that, different numbers of labeled stem cells were implanted into cartilage defects that were created in pig knee joint specimens. First, a relatively low number of dual-labeled cells (4 × 10^6^ cells / 3.7 × 10^6^ Bq activity) were implanted and imaged by clinical PET/MRI (Supplementary Fig. 9). MRI could not clearly distinguish between the labeled (red arrow) and unlabeled stem cells (white arrow) since no hypointense signal could be observed in the cartilage defects (Supplementary Fig. 9a). In contrast, the PET image showed that the labeled stem cells (red arrow) had significant signal (Supplementary Fig. 9b), while unlabeled stem cells (white arrow) were not detectable. In addition, quantification of the PET image confirmed that labeled stem cells had significantly higher radioactivity than unlabeled stem cells (Supplementary Fig. 9c). These results are consistent with the fact that PET is generally more sensitive than MRI for cell tracking applications.

**Figure 5:**
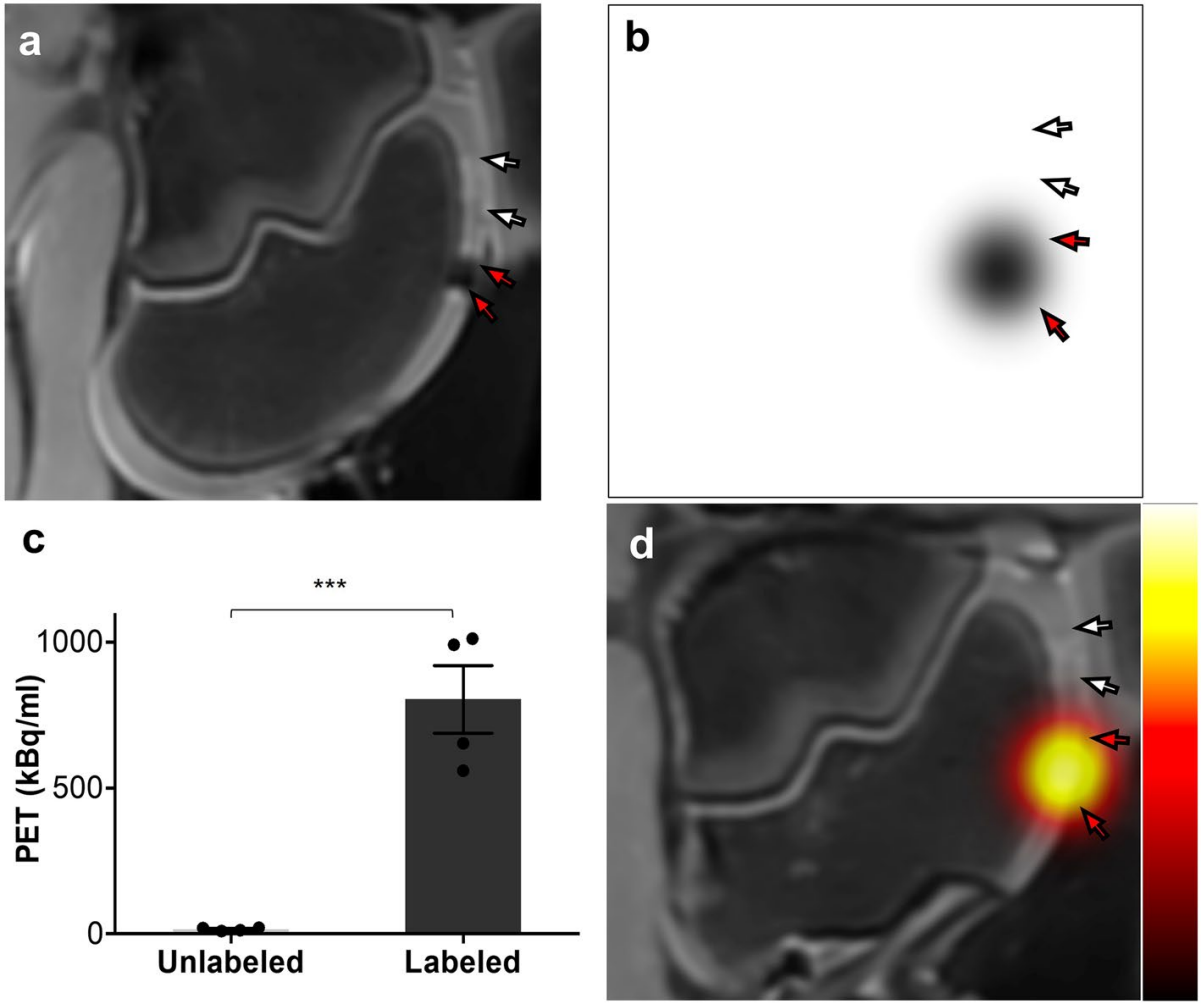
Ex vivo PET/MRI imaging of ADSCs implantation in pig knee. (**a**) MR image showing significant signal loss near labeled ADSC implant (red arrows) compared to unlabeled implant (white arrows). (**b**) Clear PET signal is also observed near the labeled ADSCs (red arrows) but not for the unlabeled implant (white arrows). (**c**) PET quantification showing significantly higher radioactivity in the labeled implant. (**d**) Fused PET/MR images showed co-localization of PET and MR signals (red arrows).

Next, we consider the transplantation of a tenfold larger number of labeled cells (60 × 10^6^ cells/7.4 × 10^6^ Bq activity). MR images clearly displayed markedly hypointense signal in cartilage defects from dual-labeled stem cells (red arrow), compared to unlabeled stem cells (white arrow; Fig. 5a). In addition, quantitative T2 mapping found significantly lower T2 relaxation times in the labeled cells (red arrows) compared to unlabeled cells (white arrows; Supplementary Fig. 10a). The labeled cell implant had an average T2 relaxation time of 102.5 ± 2.1 ms, compared to 31.0 ± 0.8 ms for the unlabeled cell implant (Supplementary Fig. 10b). Similar findings were obtained with PET. PET images demonstrated that the dual-labeled cells (red arrow) had clear focal signal, while unlabeled cells (white arrow) were not detectable (Fig. 5b). This finding was confirmed by additional quantification of the images (Fig. 5c). Finally, simultaneous PET/MR image showed colocalization (red arrows) of PET and MRI signals in the labeled cells (Fig. 5d).

## Discussion

In this study, we investigated the use of mechanoporation using a microfluidic device for labeling stem cells with ^68^Ga-labeled MSNs. Compared to conventional passive incubation, the use of the microfluidics device resulted in faster labeling and reduced DNA damage thanks to shorter exposure to ionizing radiation. There were no detectable effects on other biological endpoints, such as proliferation and apoptosis, indicating that this approach is safe and does not affect the long-term viability of the cells. Additionally, MSN-mechanoporated cells displayed favorable toxicity profile with relatively minor early acute effects. In addition, our previous results investigated the rate of efflux and its mechanism, the physicochemical characteristics of the MSNs, and the effect of the procedure on stemness and differentiation of the labeled cells^10,14^. In previous data regarding the efflux of ^68^Ga-MSN labeled cells^10^, iTLC measurements showed that radiotracer efflux by cells was composed primarily of free ^68^Ga radioisotope. The accumulation of the effluxed free ^68^Ga in the body should be considered to eliminate the possibility for misinterpretation of the images. Finally, the labeled stem cells with MSNs were successfully imaged using PET/MRI in an ex vivo model of stem cell transplantation. Given the growing clinical availability of ^68^Ga and the generally good biocompatibility of silica nanoparticles, this approach provides a rapid and straightforward method for verifying the implantation of therapeutic cells post-surgery.

Our results in Fig. 3 show that the MSNs were successfully labeled with clinical-grade ^68^Ga. MSNs have attractive properties when used as nanometer-sized delivery vehicles to carry small molecules compounds and drugs into cells and tissues, including high stability, large surface area, and tunable pore size^18,19^. In this experiment, MSNs efficiently delivered ^68^Ga radioisotope into stem cells, enabling transfer of up to 0.5 Bq/nanoparticle^10^. These nanoparticles also have excellent biocompatibility. Silica is “Generally Recognized As Safe” by the FDA for use as food additives and cosmetics^23,24^. Our study confirms the excellent biocompatibility of this system with respect to ADSCs (Fig. 4 and Supplementary Fig. 3). Therefore, our system using MSNs could be a good candidate for use in clinical trials of cell-based therapy. The approach may be particularly valuable for regenerative medicine procedures, where cells are ideally harvested from the patient and transplanted to a new site in a single surgery^1,2,25^. Our approach using mechanoporation could be very helpful for this process, because other methods for cell labeling require considerably longer time to label cells with imaging biomarkers. In addition, in the case of cell-based immunotherapy, our system could provide valuable information for tracking and monitoring therapeutic CAR-T cells for clinically application^26,27^. Finally, given the availability of inexpensive intraoperative gamma cameras^28^, the labeled cells could also be imaged in the operating room to verify the implant prior to releasing the patient.

Results from cell uptake studies (Fig. 3b–d) show that microfluidic-based mechanoporation is more efficient than conventional passive labeling. Mechanopration labeling takes less than 15 min with the current microfluidics device, which contains 5 parallel channels and is comparable in efficiency to > 30 min passive incubation. It is important to note that the labeling procedure itself only requires a few seconds, but due to the finite flow rate of the device (0.5 ml/min), it takes considerably longer to label millions of cells. Devices with many more parallel channels could be developed to achieve greater throughput and even faster labeling.

In contrast, classical transfection techniques by incubation using lipofectamine are slow and are better suited for adherent cells^3,6,29^. Alternative labeling approaches using electroporation have a detrimental effect on cell viability and may require additional processing to separate viable from non-viable cells before transplantation^30^. Additional techniques such as fluid shear stress^31^ and cavitation induction^32^ have low efficiency and impaired cell viability.

The microfluidics mechanoporation system presented here has many advantages such as fast labeling, high efficiency and high cell viability, all of which are required to enable cell harvest, cell labeling, and cell transplantation to be carried out in one simple procedure. The system is designed to repetitively compress the treated cells and thereby induce convectively driven volume exchange through transient cellular pores. TEM experiments show that the size of the MSNs taken up into the cells varies from 50 to 500 nm, with most of the particles having a size near the average value of about 200 nm, matching the original distribution of the MSNs. A small number of MSNs larger than 500 nm were observed inside of cells. Due to the expected size of the pores, it is unlikely that these particles were delivered directly to the cytosol via mechanoporation. Instead, the intracellular delivery process of these particles is likely due to endocytosis followed by endosomal escape and/or maybe low amount of direct cytosolic delivery. In addition, lipofectamine is likely to contribute to the transport of MSNs into cells, suggesting that, perhaps, mechanoporation allows for better attachment of the lipid-coated MSNs onto the negatively charged membrane prior to endocytosis. Cytosolic release could then take place through the endosomal escape properties of lipofectamine^33^. Thus, although mechanoporation has been used in other applications to induce convective transport through transient pores, in this study, we cannot unambiguously distinguish between mechanoporation-driven MSN transport and lipofectamine-facilitated uptake.

In addition, we had previously demonstrated in vivo tracking of single cells labeled with ^68^Ga-MSNs through passive transport^10^. Potentially, mechanoporation would be a powerful method for this application. Given the short half-life of ^68^Ga (67 min), rapid mechanoporation would save crucial time to further enhance cell labeling efficiency. Additionally, higher labeling efficency can be achieved with mechanoporation by flowing the cells through the device multiple times. Other radiometals, such as ^89^Zr, could also be used to label MSNs for long-term follow-up, as previsouly demonstrated^10^.

In conclusion, MSN-assisted microfluidic mechanoporation is a new approach for labeling cells for in vivo cell tracking applications. Its notable features are the high labeling efficiency that enables greater than 5 Bq per cell and the fast processing time, although further studies are required to better understand the mechanism of nanoparticle uptake. The method could be used in regenerative medicine, since it is fast enough to be incorporated into the surgical workflow, providing real-time feedback on the implantation of the cells at the site of interest. In oncology, the approach could be used to study the early seeding of cancer cells during metastasis or to optimize schedules and routes of administration of cell-based cancer immunotherapies^34^.

## Supplementary Information

Supplementary Information.
