# Supplementary material for: Mechanoporation enables rapid and efficient radiolabeling of stem cells for PET imaging
Source: Sci Rep. 2022 Feb 22;12:2955. doi: 10.1038/s41598-022-06938-6 (PMC8863797; doi:10.1038/s41598-022-06938-6)
Supplement: Supplementary file 1 — Supplementary Information. [file 41598_2022_6938_MOESM1_ESM.docx]

**Mechanoporation enables rapid and efficient radiolabeling of stem cells for PET imaging**

**Kyung Oh Jung, ^1,2,3¥^  Ashok Joseph Theruvath, ^4^ Hossein Nejadnik, ^4,7^ Anna Liu, ^5^ Lei Xing, ^1,2^**

**Todd Sulchek, ^5^ Heike E. Daldrup-Link, ^4,6^ Guillem Pratx^1,2¥^**

*^1^ Department of Radiation Oncology, Stanford University, CA, 94305, USA*

*^2^ Molecular Imaging Program at Stanford (MIPS), Stanford University, Stanford, CA, 94305, USA.*

*^3^ Department of Anatomy, College of Medicine, Chung-Ang University, Seoul, Korea.*

*^4^ Department of Radiology, Molecular Imaging Program at Stanford, Stanford University, CA, 94305, USA*

*^5^ Department of Biomedical Engineering, Georgia Institute of Technology, Atlanta, GA, 30332, USA*

*^6^ Department of Pediatrics, Stanford University, CA, 94305, USA*

*^7^ Department of Radiology, University of Pennsylvania, PA, 19104, USA*

**First author:** Kyung Oh Jung

Department of Anatomy

College of Medicine

Chung-Ang University

Seoul, Korea.

Tel: +82 (10) 3919-5576; Fax: +1 (650) 723-7254; e-mail: [kojung@cau.ac.kr](mailto:kojung@cau.ac.kr)

**^¥^ Corresponding author:** Guillem Pratx

Department of Radiation Oncology

Division of Medical Physics

Stanford University School of Medicine

Stanford, CA, USA.

Tel: +1 (650) 724-9829; Fax: +1 (650) 723-7254; e-mail: [pratx@stanford.edu](mailto:pratx@stanford.edu)

**^¥^ Corresponding author:** Kyung Oh Jung

Department of Anatomy

College of Medicine

Chung-Ang University

Seoul, Korea.

Tel: +82 (10) 3919-5576; Fax: +1 (650) 723-7254; e-mail: [kojung@cau.ac.kr](mailto:kojung@cau.ac.kr)


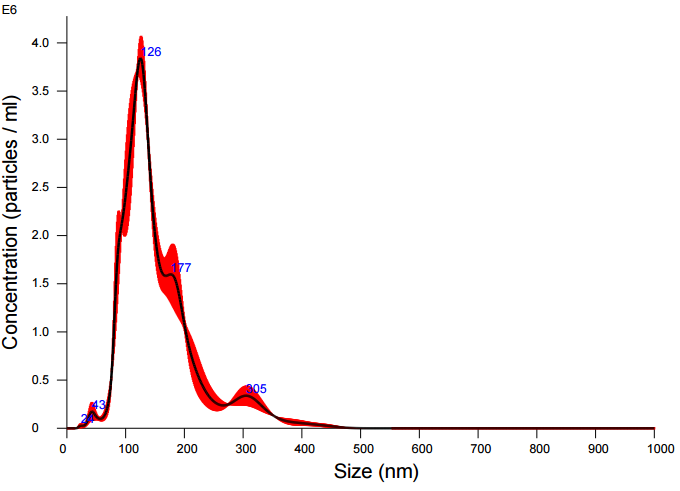


**Supplementary Figure 1. Size distribution of MSNs.** The size of MSNs was measured by Nanoparticle Tracking Analysis (Malvern Nanosight).


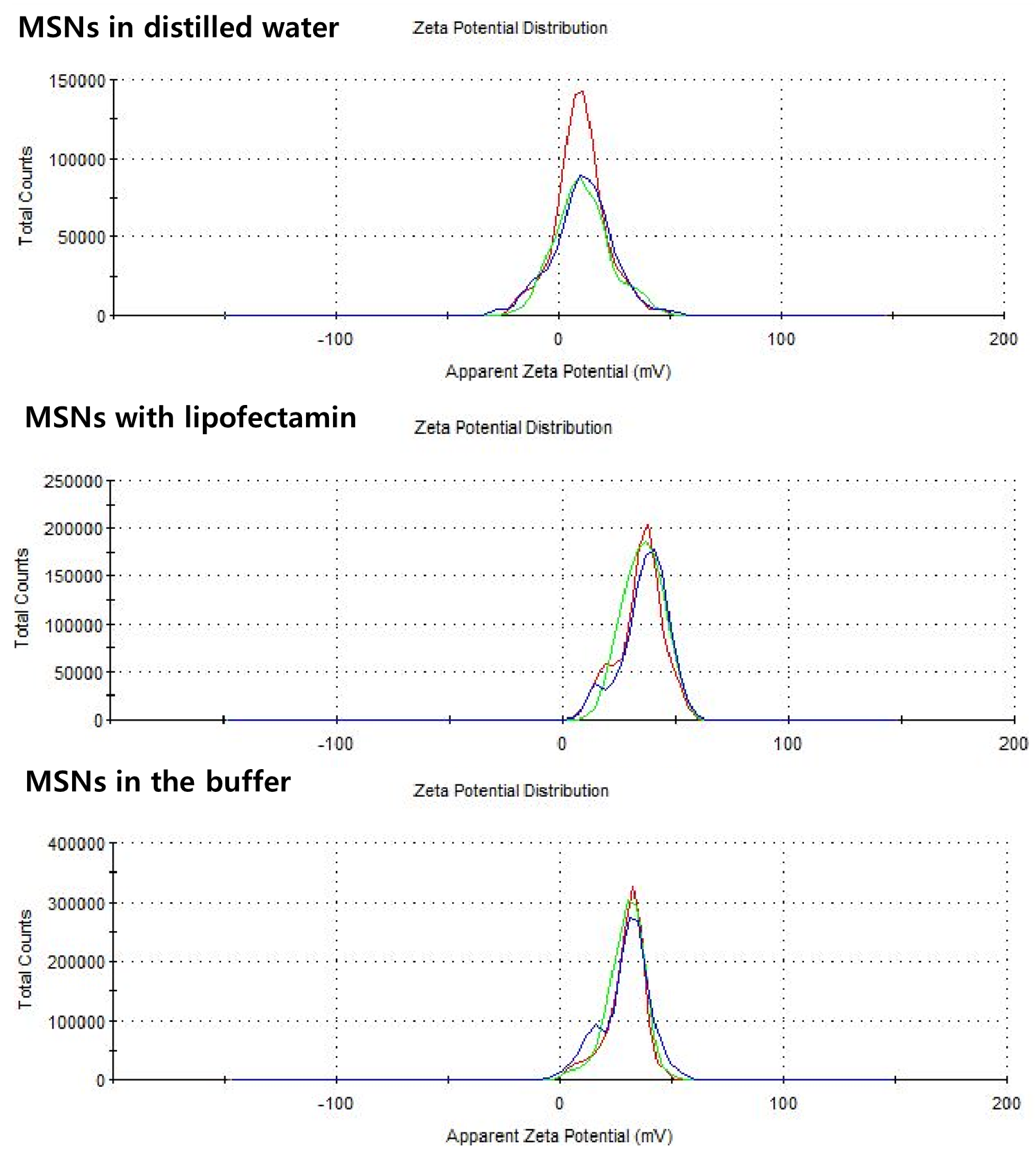


**Supplementary Figure 2. Zeta potential of MSNs.** The zeta potential of MSNs in distilled water and FACS buffer was measured by dynamic light scattering (DLS).


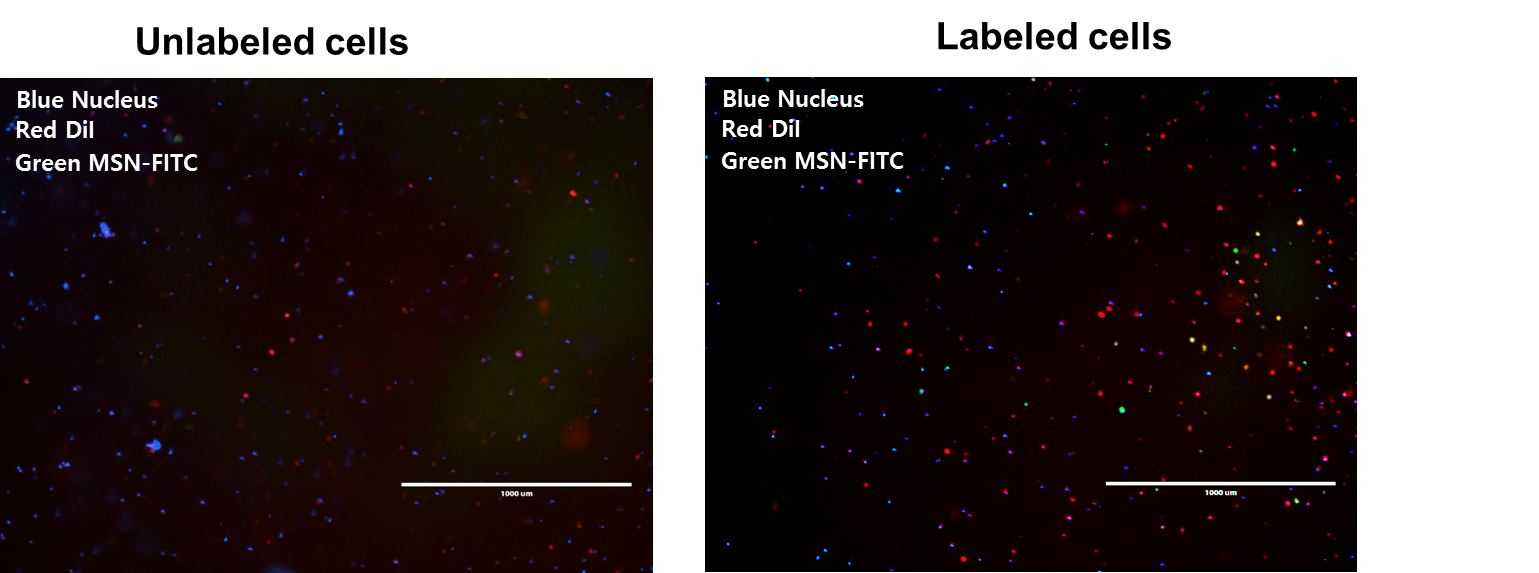


**Supplementary Figure 3. Efficiency of cell labeling by mechanoporation assessed by fluorescence imaging.** Green fluorescence indicates FITC-labeled MSN transport into cells by mechanoporation.


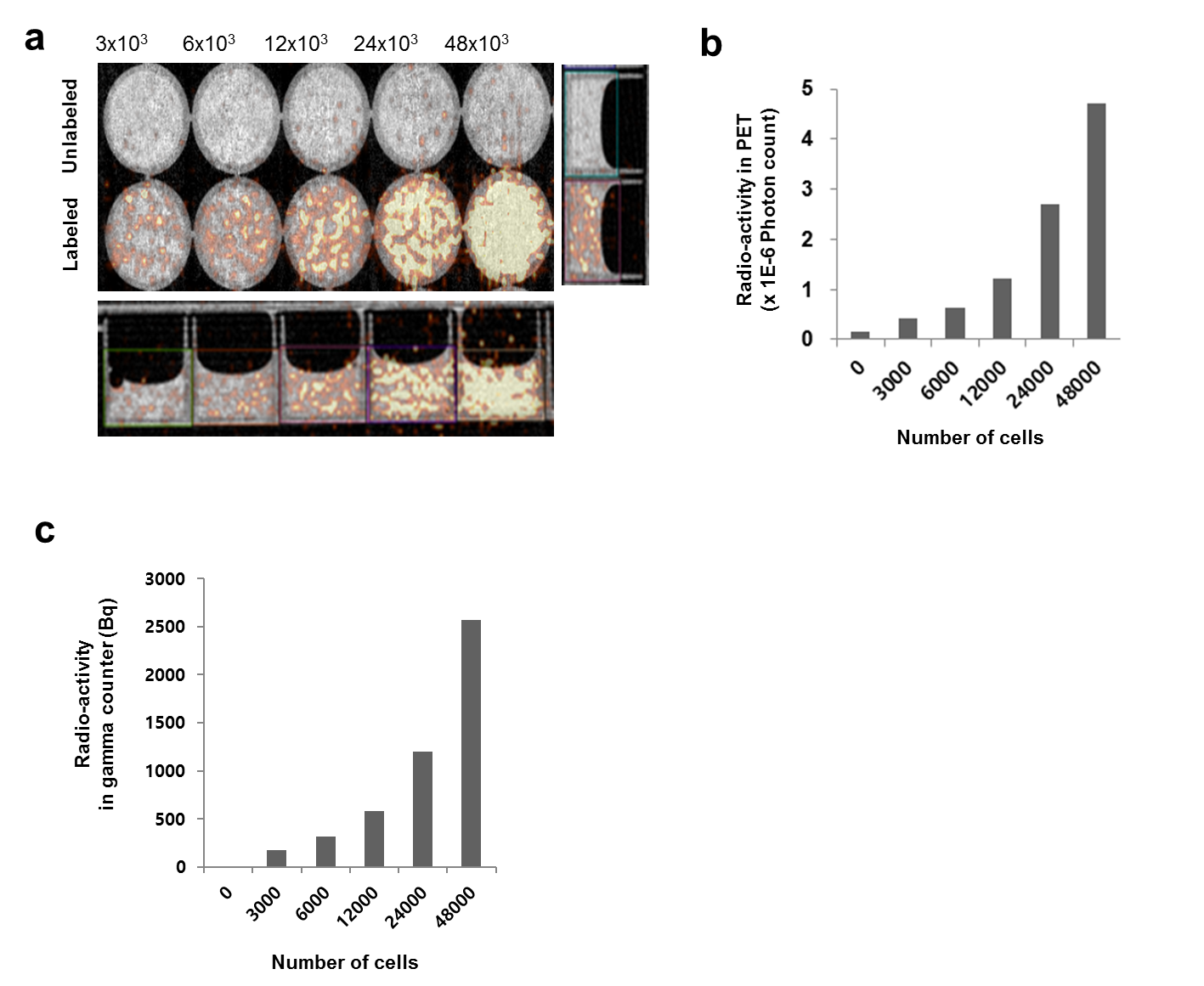


**Supplementary Figure 4. Cell labeling with ^68^Ga-MSNs by mechanoporation.** (a) PET images showing increasing signal with increasing cell number. (b) Quantitative region-of-interest analysis of PET images. (c) Radiolabeling efficiency measured by gamma counting.


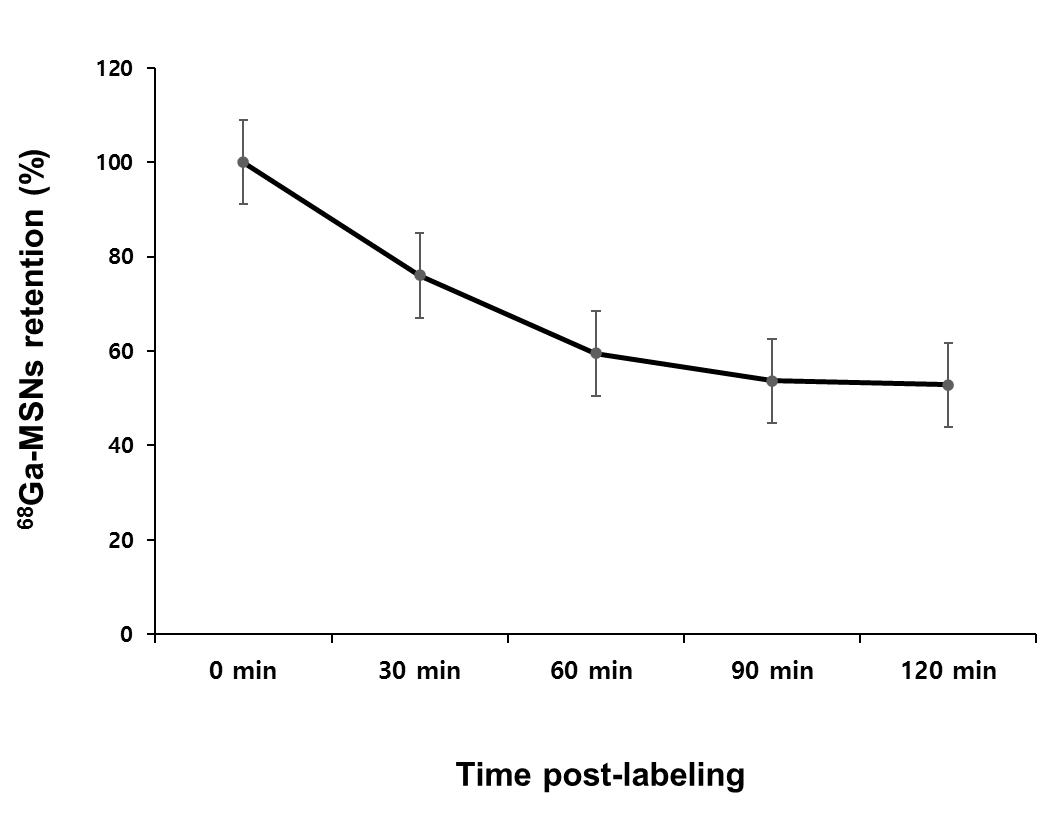


**Supplementary Figure 5. Radiotracer efflux from labeled cells after cell labeling by mechanoporation.** Gamma counting measurements found that radiotracer efflux from the labeled cells reached about 50% after 2 h.


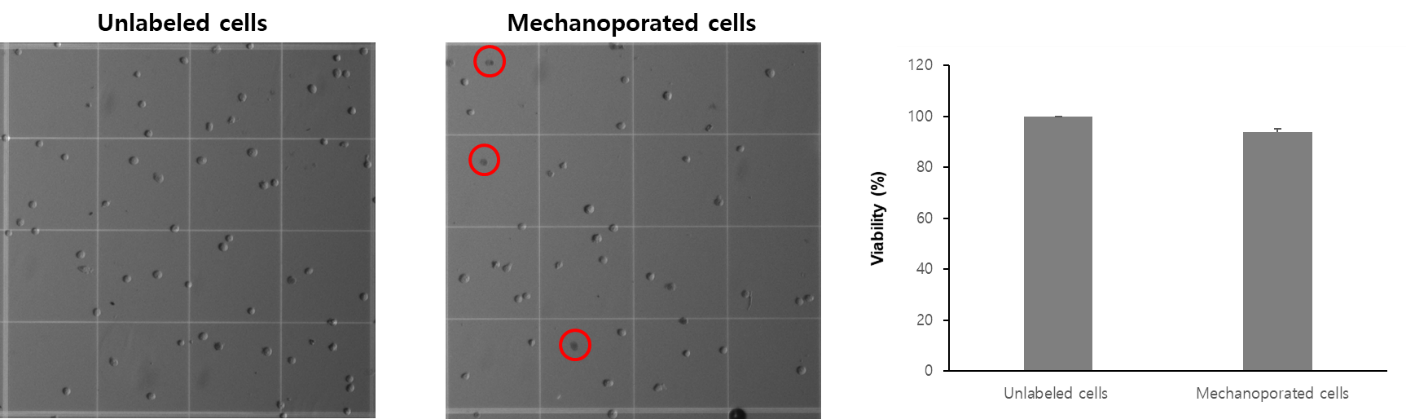


**Supplementary Figure 6. Acute toxicity induced by mechanoporation.** Trypan blue staining was performed in untreated cells and mechanoporated cells 2 hours after mechanoporation.


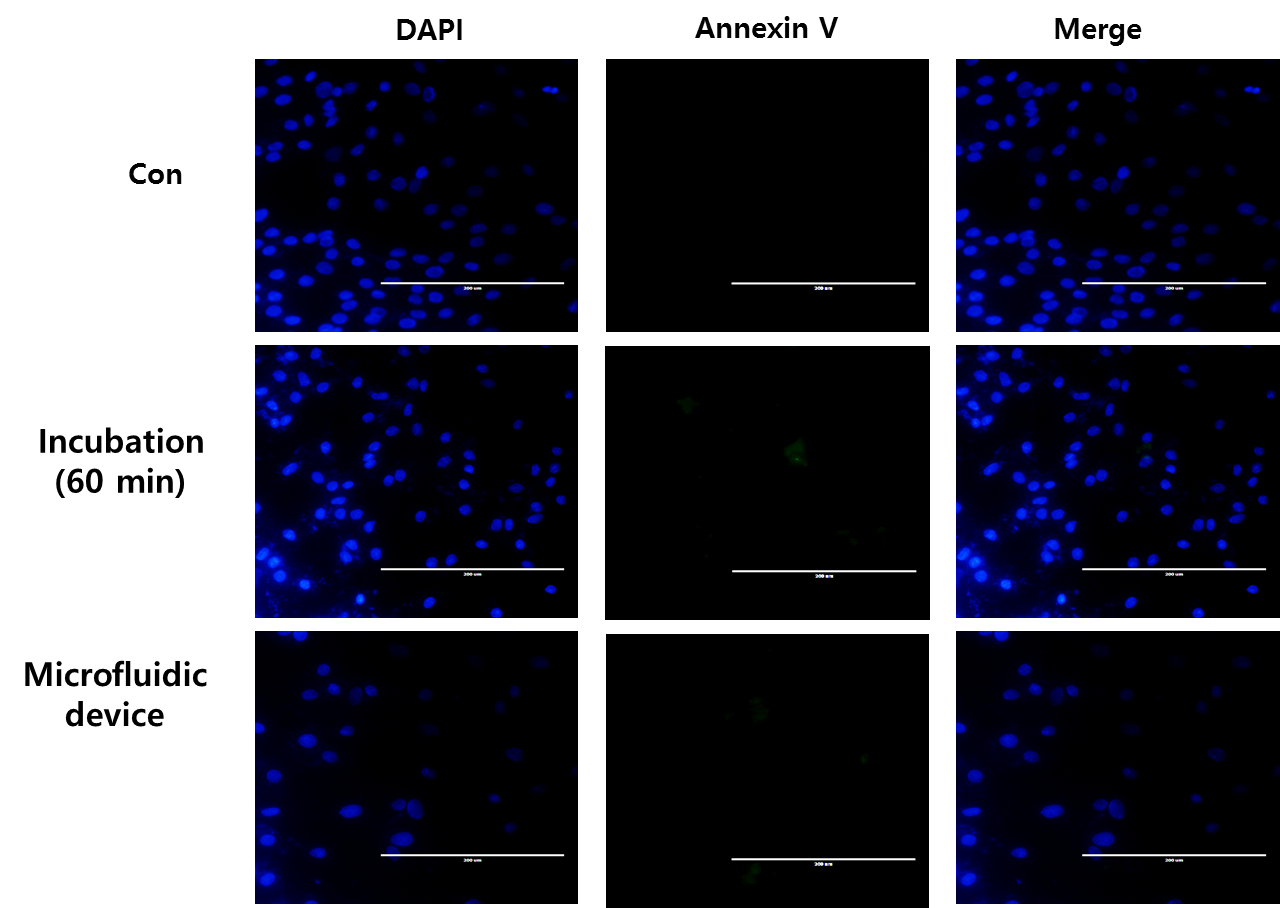


**Supplementary Figure 7. Apoptosis assay for cell biocompatibility.** Apoptosis assay showed no significant difference between unlabeled and labeled cells.


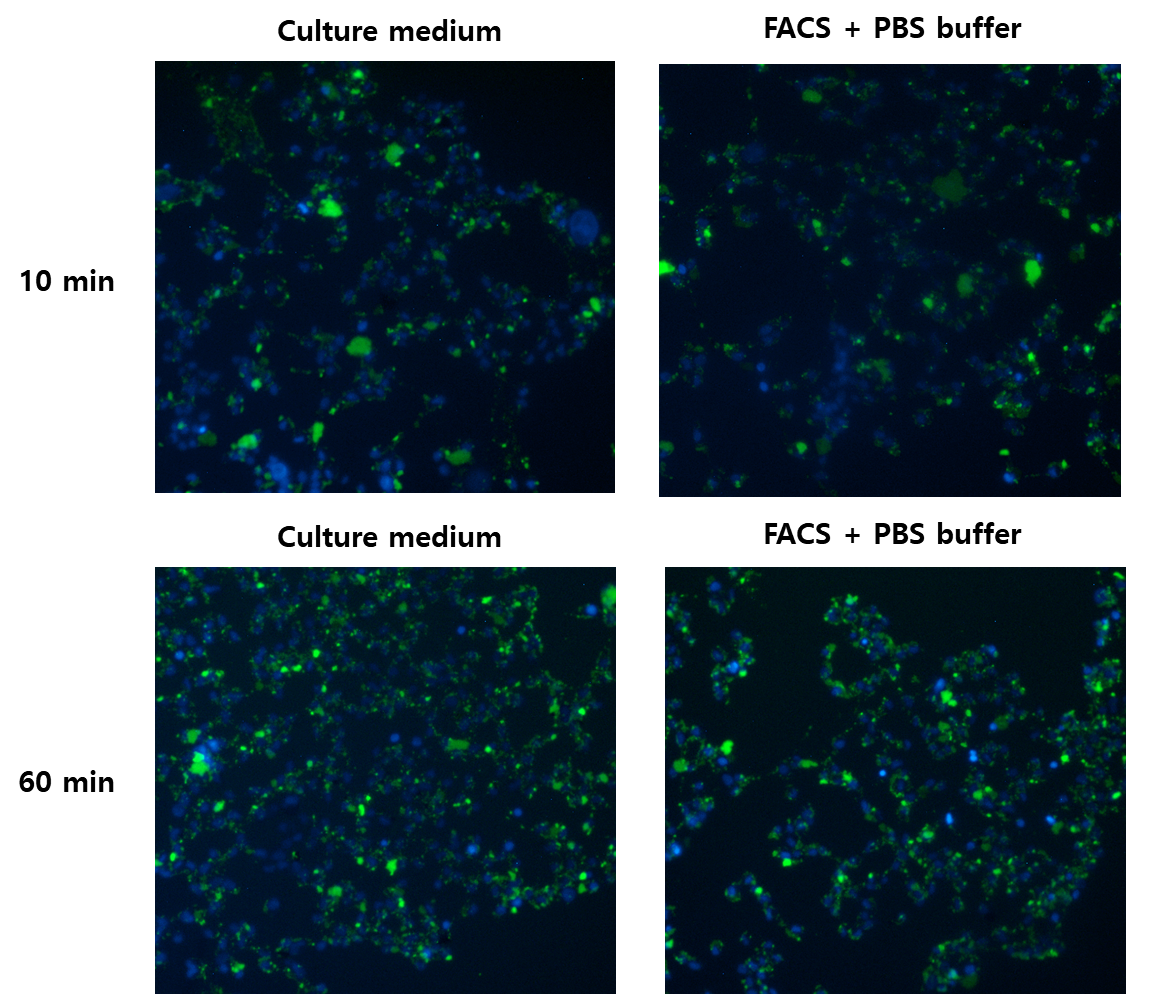


**Supplementary Figure 8. Comparison of incubation buffer.** Cells were labeled with passive incubation (10 & 60 min) in culture medium and FACS buffer. There were no visible difference between the two buffer conditions.

**
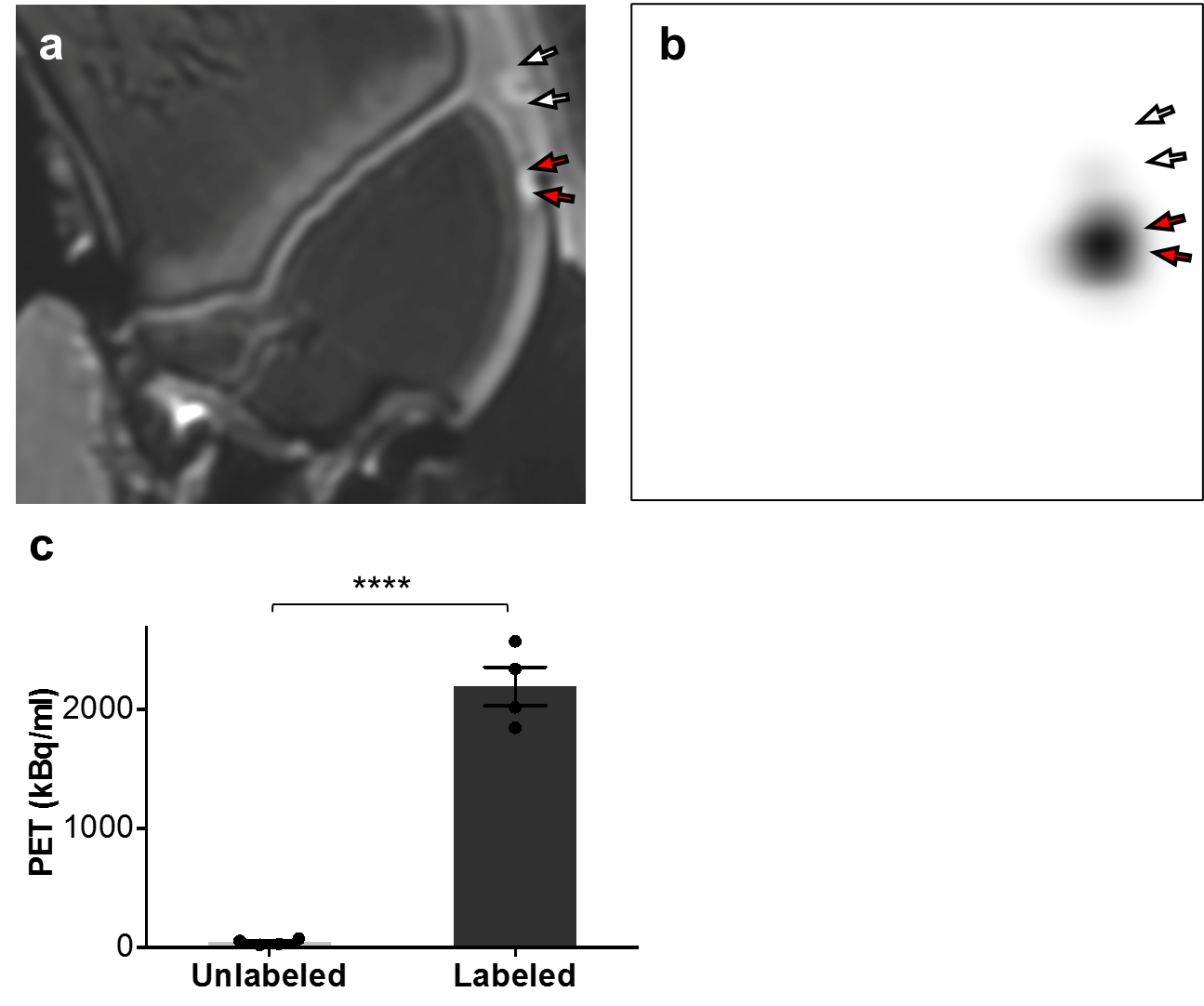
**

**Supplementary Figure 9. Ex vivo PET/MRI imaging of a small number (4×10^6^ cells) of ADSCs, implanted in pig knee.** (a) MRI image in cartilage defects showing no distinguishable hypointense signals between labeled (red arrow) and unlabeled ADSCs (white arrow) (b) Corresponding PET image clearly highlighting the labeled ADSCs (red arrows). (c) Quantitative region-of-interest analysis (*** P<0.0001).


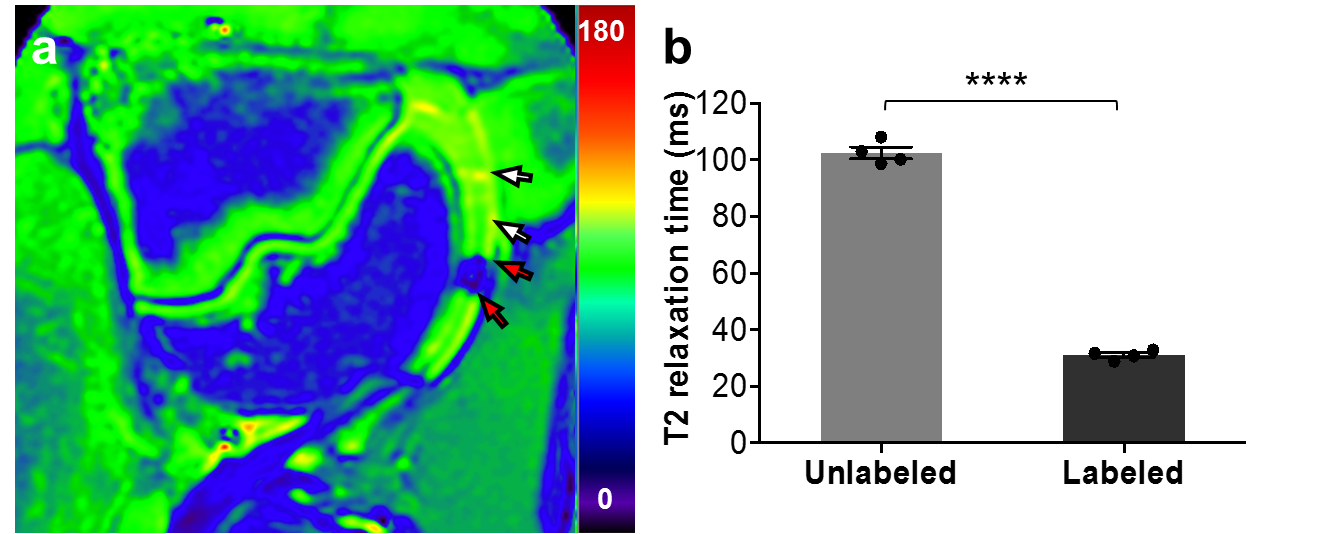


**Supplementary Figure 10. T2 mapping and MRI quantification (60×10^6^ ADSCs per implant).** (a) T2 mapping showing significantly lower T2 relaxation times in labeled implanted cells (red arrows) than unlabeled cells (white arrows). b) Quantification of MRI images. (**** P < 0.0001).
